# Hemoglobin, Albumin, Lymphocyte, and Platelet (HALP) score as a biomarker for phase differentiation in Peyronie’s Disease: a retrospective cohort study

**DOI:** 10.1186/s12610-025-00269-y

**Published:** 2025-06-05

**Authors:** Türker Soydaş, Selman Ünal, Halil Uzundal

**Affiliations:** 1 Department of Urology, Ankara Etlik City Hospital, Ankara, Türkiye; 2 Department of Urology, Urgup State Hospital, Nevsehir, Türkiye; 3 Department of Pediatric Urology, Ankara Bilkent City Hospital, Ankara, Türkiye

**Keywords:** Peyronie’s disease, HALP score, Inflammatory indices, Fibrosis, Biomarker, Maladie de La Peyronie, Score HALP, Indices inflammatoires, Fibrose, Biomarqueurs

## Abstract

**Background:**

Peyronie’s disease progresses through acute (inflammatory) and chronic (fibrotic) phases. Objective biomarkers for phase differentiation are lacking. Neutrophil-to-eosinophil ratio has been evaluated as an inflammatory marker in PD and reported as an important marker in the inflammatory phase. We evaluated the Hemoglobin, Albumin, Lymphocyte, and Platelet score as a non-invasive indicator of Peyronie’s Disease phases.

**Results:**

A retrospective cohort of 216 Peyronie’s disease patients were analyzed. Blood parameters were collected at diagnosis (acute phase) and follow-up (chronic phase) in 112 patiens. Hemoglobin, Albumin, Lymphocyte, and Platelet scores were compared using receiver operating characteristic curves and paired t-tests. Hemoglobin, Albumin, Lymphocyte, and Platelet scores increased significantly from 36.8 ± 8.2 (acute) to 47.5 ± 10.4 (chronic) in 112 paired patients (*p* <0.001). Receiver operating characteristic analysis identified a cutoff of>41.2 for chronic phase detection (area under the curve = 0.83, sensitivity 76%, specificity 71%, positive predictive value 73.9%).

**Conclusion:**

The Hemoglobin, Albumin, Lymphocyte, and Platelet score is a cost-effective tool for differentiating Peyronie’s Disease phases, aiding clinical decision-making.

## Résumé

**Contexte:**

La maladie de La Peyronie progresse à travers des phases aiguë (inflammatoire) et chronique (fibrotique). Il n’existe pas de biomarqueurs objectifs pour la différenciation de phase. Le rapport neutrophiles/éosinophiles a été évalué comme un marqueur inflammatoire dans la maladie de La Peyronie, et signalé comme un marqueur important lors de la phase inflammatoire. Nous avons évalué le score d’hémoglobine, d’albumine, de lymphocytes et de plaquettes (HALP) en tant qu’indicateur non invasif des phases de la maladie de La Peyronie.

**Résultats:**

Une cohorte rétrospective de 216 patients atteints de la maladie de La Peyronie a été analysée. Les paramètres sanguins ont été collectés au moment du diagnostic (phase aiguë) et du suivi (phase chronique) chez 112 patients. Les scores d’hémoglobine, d’albumine, de lymphocytes et de plaquettes ont été comparés à l’aide de courbes caractéristiques de fonctionnement du récepteur et de tests t appariés. Les scores d’hémoglobine, d’albumine, de lymphocytes et de plaquettes ont augmenté de manière significative, passant de 36,8 ± 8,2 (aiguë) à 47,5 ± 10,4 (chronique) chez 112 patients appariés (*p* <0,001). L’analyse des caractéristiques de fonctionnement du récepteur a permis d’identifier un seuil de >41,2 pour la détection de phase chronique (aire sous la courbe = 0,83, sensibilité 76 %, spécificité 71 %, valeur prédictive positive 73,9 %).

**Conclusions:**

Le score d’hémoglobine, d’albumine, de lymphocytes et de plaquettes (HALP) est un outil rentable pour différencier les phases de la maladie de La Peyronie, et pour aider à la prise de la décision clinique.

## Introduction

Peyronie’s disease (PD) is a connective tissue disorder characterized by the formation of fibrous plaques within the tunica albuginea of the penis, leading to penile curvature, pain, and erectile dysfunction. The disease progresses through two distinct phases: an acute inflammatory phasemarked by localized inflammation, pain, and plaque formation, and a chronic fibrotic phase characterized by stable plaques, calcification, and structural deformity [1]. During the acute phase, inflammatory cytokines such as TGF-β1 and IL-6 drive fibroblast proliferation and collagen deposition, while the chronic phase involves remodeling of the extracellular matrix and stabilization of fibrotic plaques [2]. The gold standard treatment method for the disease is surgery, but surgery is recommended in the chronic phase, while symptomatic treatments (e.g. traction devices, ESWT) and anti-inflammatory drugs are at the forefront in the acute phase [3].

Current diagnostic methods rely on clinical history, physical examination, and imaging modalities like penile ultrasound. However, these approaches lack objectivity in differentiating early inflammatory activity from late-stage fibrosis. Biomarkers that reflect systemic inflammation and nutritional status could provide a non-invasive, cost-effective tool for phase stratification. The Hemoglobin, Albumin, Lymphocyte, and Platelet (HALP) [4] score, initially developed as a prognostic marker in oncology and inflammatory diseases, integrates four parameters:

Hemoglobin: Reflects anemia of chronic disease, often seen in inflammatory states. Albumin: A negative acute-phase reactant that decreases during inflammation. Lymphocyte: Represents immune response modulation. Platelet: Elevated in chronic inflammation and tissue repair.

Recent studies, have highlighted the role of systemic immune-inflammation (e.g. neutrophil-to-eosinophil ratio) indices in PD, suggesting that composite biomarkers may better capture the multifactorial nature of disease progression [5–7]. In this study, the usability of the HALP score, a systemic inflammation assessment that has not been evaluated before in PD, in distinguishing between acute and chronic PD stages was evaluated.

## Methods

### Study design

This retrospective cohort of 216 PD patients were analyzed. Blood parameters were collected at diagnosis (acute phase). Among these, 112 patients returned for follow-up during the chronic phase and were included in paired comparisons. Patients were included if they met the following criteria; diagnosis of PD with confirmed by physical examination (palpable penile plaque) and penile ultrasound (plaque visualization and curvature measurement); phase stratification, acute phase defined by symptom duration <12 months, presence of penile pain, and plaque instability on ultrasound and chronic phase defined by symptom duration ≥12 months, absence of pain, and stable plaque characteristics [3].

#### Blood tests

Availability of complete blood count (CBC) and albumin levels at both diagnosis (acute phase) and follow-up (chronic phase).

Exclusion criteria were active infections (e.g., urinary tract infections), autoimmune diseases (e.g., rheumatoid arthritis), or malignancy, use of anti-inflammatory medications (e.g., NSAIDs, colchicine) within 4 weeks of blood sampling, incomplete clinical or laboratory records. Blood samples were collected under standardized conditions (fasting, morning hours). Parameters included:

- Hemoglobin (g/dL): Measured via automated hematology analyzer.
- Albumin (g/dL): Assessed using bromocresol green method.
- Lymphocyte, Platelet, and Leukocyte counts (×10^3^/µL): Derived from CBC.

The HALP score was calculated as [7]:

$$\text{HALP}=\left[\text{Hemoglobin}\left(\text{g}/\text{dL}\right)\times\text{Albumin}\left(\text{g}/\text{dL}\right)\times\text{Lymphocyte}\;\text{count}\right]/\text{Platelet count})$$

### Statistical analysis

Data were analyzed using SPSS v25 (IBM Corp.). Continuous variables were expressed as mean ± standard deviation (SD) or median (interquartile range), while categorical variables were presented as percentages. Paired t-tests compared acute and chronic-phase parameters. Receiver operating characteristic (ROC) curves determined optimal HALP cutoffs for phase differentiation, with area under the curve (AUC) values quantifying diagnostic accuracy. A two-tailed *p*-value <0.05 was considered statistically significant.

## Results

### Demographics and clinical characteristics

The cohort comprised 216 PD patients classified as acute-phase but 112 (43%) patients continued to follow-up as chronic-phase patients. 148 patients were excluded because they did not continue their follow-up. Demographic characteristics are summarized in Table 1. No significant differences were observed in age, smoking status, or comorbidities between phases. While nutritional effects such as cachexia and sarcopenia that may develop in oncological patient groups may affect the HALP score [7], we think that nutritional status may have a minimal effect in our patient group because it is a single patient group with acute and chronic phase follow-up of the patients.

**Table 1:** Demographic and clinical characteristics

Parameter	Acute Phase (*n*=112)	Chronic Phase (*n*=112)	*p*-value
Age (years) mean ± (SD)	52 ± 9	53 ± 8	0.32
Disease Duration (months) mean ± (SD)	4.2 ± 1.5	16.1 ± 4.3	<0.001
Smoking (%)	44	43	0.89
Hypertension (%)	28	30	0.75
Diabetes Mellitus (%)	15	17	0.68

Legend: Data presented as mean ± standard deviation (SD) or percentage (%). Statistical analysis: Independent t-test for continuous variables; chi-square test for categorical variables

**Fig. 1:**
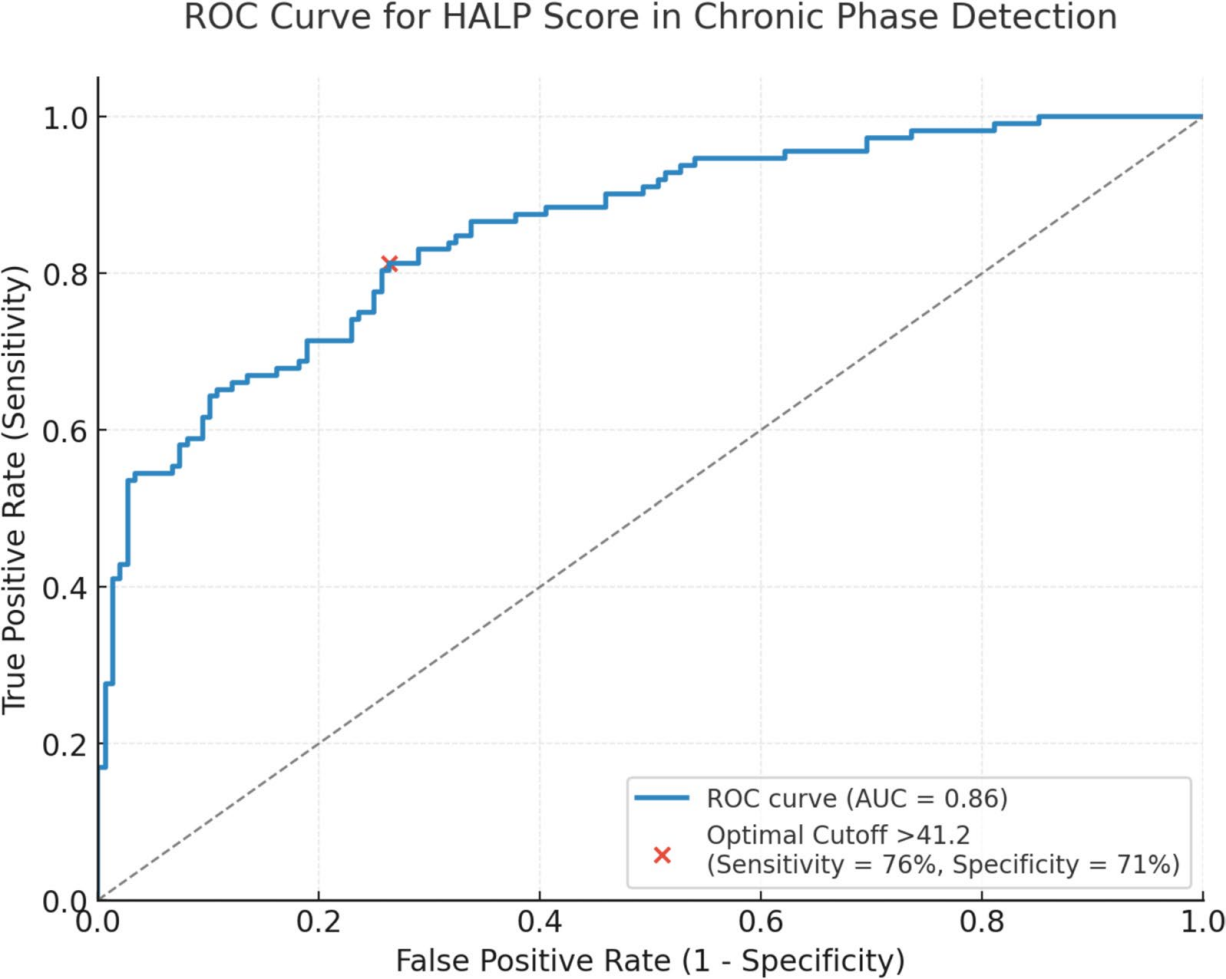
ROC curve for HALP Score. Legend: Receiver operating characteristic (ROC) curve evaluating the Hemoglobin, Albumin, Lymphocyte, and Platelet (HALP) score’s ability to differentiate chronic-phase Peyronie’s disease (PD). The dashed line indicates the optimal cutoff value (>41.2), achieving a sensitivity of 76% and specificity of 71%. The area under the curve (AUC) is 0.83 (95% confidence interval: 0.76–0.89), demonstrating strong diagnostic performance. Statistical analysis was performed using ROC curve methodology. Abbreviations: HALP, Hemoglobin, Albumin, Lymphocyte, Platelet; PD, Peyronie’s disease; AUC, area under the curve

Table 2 compares acute and chronic-phase blood parameters. Hemoglobin, albumin, lymphocyte, platelet, and leukocyte counts showed no significant differences between phases (*p* > 0.05). In contrast, the HALP score increased significantly from 36.8 ± 8.2 (acute) to 47.5 ± 10.4 (chronic) in these 112 patients (*p*< 0.001). ROC curve analysis demonstrated strong diagnostic accuracy for HALP in chronic-phase identification (Fig. 1). The optimal cutoff of >41.2 yielded 76% sensitivity and 71% specificity, PPV 73.9% (AUC = 0.83, 95% CI: 0.76–0.89). According to these results, 85 patients out of 112 patients in the cohort were evaluated as true positive.

**Table 2:** Comparison of blood parameters and HALP score

Parameter	Acute Phase (*n*=148)	Chronic Phase (*n*=112)	*p*-value
Hemoglobin (g/dL)	13.1 ± 1.3	14.0 ± 1.2	0.12
Albumin (g/dL)	3.7 ± 0.5	4.3 ± 0.4	0.07
Lymphocyte (×10^3^/µL)	1.8 ± 0.6	2.0 ± 0.5	0.15
Platelet (×10^3^/µL)	245 ± 62	230 ± 58	0.22
Leukocyte (×10^3^/µL)	7.2 ± 1.8	6.9 ± 1.6	0.35
HALP Score	36.8 ± 8.2	47.5 ± 10.4	<0.001

Statistical analysis: Paired t-test

Legend: *HALP* Hemoglobin, Albumin, Lymphocyte, Platelet score, *CBC* Complete blood count

## Discussion

This study reveals that while hemoglobin, albumin, and inflammatory cell counts (lymphocyte, platelet, leukocyte) exhibited no statistically significant differences between phases, the HALP score showed a substantial increase during the chronic phase (*p* < 0.001). This highlights HALP’s unique advantage as a composite biomarker that integrates nutritional and inflammatory pathways, enabling it to effectively differentiate between acute and chronic PD phases—a distinction unattainable with isolated parameters. The significant increase in HALP during the chronic phase (*p* <0.001) aligns with the resolution of inflammation and stabilization of fibrotic plaques.

The gold standard treatment for penile deformities resulting from Peyronie’s disease is surgery [8]. However, this does not mean that all patients require surgical treatment. If the deformities do not affect the patient’s sexual intercourse, surgical treatment is not necessary. The fact that 148 of the 216 patients in this cohort did not re-apply in the chronic phase suggests that they did not need or desire surgical treatment.

### Pathophysiological insights

#### Hemoglobin and albumin

Acute-phase reductions likely reflect anemia of chronic disease and cytokine-driven hypoalbuminemia. Chronic-phase improvements correlate with diminished inflammatory activity. It was important that there was no statistical difference in the follow-up of the patients in terms of age, smoking and comorbidity, as it could affect the complete blood count parameters.

#### Lymphocyte and platelet counts

The lack of significant changes suggests that PD’s chronic phase involves localized, rather than systemic, immune dysregulation.

These findings align with literature, that reported associations between systemic immune-inflammation indices and PD severity [5]. However, unlike neutrophil-to-eosinophil ratios, the HALP score integrates nutritional status, providing a broader reflection of disease burden.

### Clinical implications

The HALP score offers several clinically actionable insights for managing PD. First, its ability to objectively differentiate acute inflammatory and chronic fibrotic phases addresses a critical gap in current diagnostic practices, which rely heavily on subjective clinical assessments. Early identification of the acute phase (via low HALP scores) could prompt timely initiation of anti-inflammatory therapies to mitigate plaque progression and reduce penile curvature. Conversely, a rising HALP score during follow-up may signal transition to the chronic phase, guiding clinicians to prioritize surgical interventions (e.g., plication or grafting) in patients with stable plaques and significant deformity, thereby optimizing functional outcomes.

Additionally, the HALP score’s cost-effectiveness and routine availability in standard blood panels make it an accessible tool for serial monitoring. For instance, in resource-limited settings where advanced imaging is unavailable, tracking HALP trends could serve as a surrogate marker for disease activity, enabling dynamic treatment adjustments. Furthermore, integrating HALP with imaging modalities like ultrasound elastography may enhance prognostic accuracy, aiding in personalized patient counseling.

### Limitations of the study

Limitations; retrospective design, single center cohort and lack of longitudinal data on HALP trends post-surgery.

## Conclusion

In summary, the HALP score bridges the gap between pathophysiology and clinical practice, offering a practical framework for phase-specific management. Its adoption could reduce unnecessary treatments in stable patients, lower healthcare costs, and improve patient satisfaction through tailored therapeutic strategies and may enhance personalized management, optimizing the timing of medical and surgical interventions. Prospective multicenter studies should validate its utility and correlate it with imaging biomarkers.

## Data Availability

Data is hidden in a double protected USB memory stick

## Abbreviations

PD: Peyronie’s disease
HALP: Hemoglobin, Albumin, Lymphocyte, Platelet
ROC: Receiver operating characteristic
AUC: Area under the curve
PPV: Positive predictive value
CBC: Complete blood count
TGF-β1: Transforming growth factor beta 1
IL-6: Interleukin 6
